# Supplementary material for: Adipose-Derived Stromal Cells within a Gelatin Matrix Acquire Enhanced Regenerative and Angiogenic Properties: A Pre-Clinical Study for Application to Chronic Wounds
Source: Biomedicines. 2023 Mar 22;11(3):987. doi: 10.3390/biomedicines11030987 (PMC10046849; doi:10.3390/biomedicines11030987)
Supplement: Supplementary file 1 [file biomedicines-11-00987-s001.zip › biomedicines-2273784-supplementary.pdf]

SUPPLEMENTARY MATERIALS

Supplementary Materials and Methods

Rat Model of ischemic wound

The model of ischemic wound in the rat was performed as previously described (Kallmeyer et al., 2020, Tobalem et al., 2015). Briefly, Wistar female rats of 250-300 g were pre-anesthetized by inhalation of isoflurane 5%, and anesthetized at the dose of 2%. Hairs were removed from the inguinal region using a mechanical shaver. All surgical procedures were performed under an operating microscope. Through a longitudinal incision made in the upper part of the left thigh, the external iliac and femoral arteries were dissected free along their entire length, from the common iliac to the saphenous artery, and one cm-length artery was removed. Immediately after the arterial resection, a wound was created on the dorsal aspect of the feet in all animals by removing a full-thickness skin area of 1.2 × 0.8 cm. Treatments were applied a day after the surgery. Rat ASCs were generated from the inguinal non-ischemic fat of control rats two months after the induction of paw ischemia.

Flow cytometry and multipotent differentiation of ASC

Cells were incubated with fluorochrome-labeled antibodies for 30 minutes at 4°C in binding buffer (BD Biosciences), prior to analysis using a BD Accuri^{TM}-B6 flow cytometer (BD Biosciences). Antibodies were as follows: i) for human cells: mouse IgG1 anti-CD44/CD73/CD90/CD45/CD105/CD14/HLA-DR (all from Abcam); ii) for rat cells: Armenian hamster anti-rat CD29-APC (clone HMb1-1, ThermoFischer), mouse anti-rat-CD31-PE (clone TLD-3A12, BD Pharmingen), mouse anti-rat CD45-BV421 (clone OX-1, BD Pharmingen) and mouse anti-rat CD90-BB515 (clone OX-7, BD Pharmingen). Analysis was

performed on viable cells (negative for Draq7), upon exclusion of cell doublets. Cell purity was >98%.

The multipotent differentiation into adipocytes, osteocytes and chondrocytes was performed by using the Human Mesenchymal Stem Cell Functional Identification Kit (R&D Systems) according to the supplier's instructions.

Immunocytochemistry and immunofluorescence on tissue sections

ASC were cultured on glass coverslips prior to fixation with paraformaldehyde 0.5% for 15 minutes at RT. Cells were incubated overnight (o/n) at 4°C in PBS containing 0.3% Triton X-100 and 0.5% bovine serum-albumin with the following primary antibodies: mouse IgM anti-Stro-1 (Clone STRO1, Thermo scientific). Detection was achieved using an anti-mouse IgM-Alexa 555 antibody for one hour at +4°C. Cells were stained with DAPI 1μg/ml for 10 minutes prior to final washing and mounting.

For histological analyses, tissues were washed in PBS and fixed with a 4% paraformaldehyde solution for 20 minutes prior to dehydration and embedment in paraffin. Upon rehydration, slides were stained in PBS supplemented with bovine serum albumin 1%, Triton X-100 0.3% o/n at 4°C with a mouse IgG anti-CD31 (Abcam). Upon staining with anti-mouse IgG-Alexa 555 antibody, slides were counterstained with DAPI and mounted in FluorSave medium (Calbiochem). Hematoxylin & Eosin staining and Masson's trichrome staining were performed according to the standard protocol. Vessel area was computed by QuPath software as a function of CD31 staining (above a defined threshold) in at least 3 sections per condition analyzed.

Immunocytochemistry and immunofluorescence applied to osteocytes, chondrocytes and adipocytes derived from ASC was performed with the reagents of the human mesenchymal stem cell functional identification kit (RnDSystems)

Cytokine measurements

Cytokines were measured in the supernatants from ASC cultures and ASC-enriched patches using the human cytokine base kit A (R&D Systems) combined with a magnetic Luminex assay (Bio-plex 200, Biorad) according to the manufacturer's instructions.

Microarrays

Isolation of total RNA was performed by using RNeasy kit from Qiagen according to the manufacturer's instructions. RNA concentration was determined by a spectrometer (Thermo Scientific™ NanoDrop 2000) and RNA quality was verified by 2100 bioanalyzer (Agilent). Human and rat microarray was performed with the Clariom™ S Assay's for human and rat, respectively, (Thermofisher) using the Complete GeneChip® Instrument System, Affymetrix. Hierarchical clustering and principal component analysis were computed using TAC4.0.1.36 software (Biosystems) using the pheatmap package (https://cran.r-project.org/web/packages/pheatmap/index.html) with default settings. Gene Set Enrichment Analysis (GSEA), desktop version 3.0 was used to analyze the pattern of differential gene expression between the human ASC-patch and the monolayer condition. The Gene Ontology Biological Process (GOBP) gene set from the Molecular Signatures Database was used. Result of GSEA analysis were visualized for enrichment map using Cytoscape 3.8.2. Enrichment of processes and pathways within the significantly upregulated or downregulated transcripts (fold change>2, FDR<0.01) identified in the rat ASC-path compared to rat ASCs grown in monolayer was assessed using Metascape (Zhou et al., 2019). The parameters used for the analysis were as follows: Organisms: Rattus Norvegicus, Input gene set: GO Biological Process; Min Overlap: 3; P value cutoff:0.01; Min enrichment: 0.01).

Mass spectrometry

Cultured human ASC or ASC-enriched patches were incubated for 45 minutes with collagenase NB6 (Nordmark) at 0.3 U/ml, washed with a serum-free DMEM (Thermofisher) and cultured for 24h at 37°C in serum-free medium. Upon clarification of supernatants at 500 x g for 10min, proteins were precipitated, digested and peptides analyzed by nanoLC-MSMS using an easynLC1000 (Thermofisher) coupled with a Q Exactive HF mass spectrometer (Thermofisher). Database searches were performed with Mascot (Matrix Science) using the Human Reference Proteome database (Uniprot). Data were analyzed and validated with Scaffold (Proteome Software) with 1% of protein FDR and at least 2 unique peptide per protein with a 0.1% of peptide FDR.

Functional tests on skin cells (fibroblasts and keratinocytes)

Human foreskin fibroblasts (provided by Neurix SA, Plan-les-Ouates) were cultured in 24-well culture plates in DMEM culture media (4,5 g/l glucose, L-Glutamine) supplemented with 10% fetal calf serum (Gibco) and 1% penicillin-streptomycin (Gibco). Upon washing to remove eventual cell debris, fibroblasts were cultured for 18 hours in serum-free media in the presence or absence of conditioned media from ASC cultures (serum-free medium pre-incubated with cultured ASC or ASC-enriched patches) at the concentration of 720 000 cells/ cm2. The number of fibroblasts was counted by trypan blue exclusion.

Primary human keratocytes were obtained from the skin of individuals undergoing abdominoplastic surgery as previously described (Borowczyk et al., 2020). Cells were seeded onto an ASC-enriched patch and initially cultured in a submerged manner in fully-supplemented CnT-Prime Medium (CELLnTEC, Bern, Switzerland). Medium was

subsequently changed to CnT-Prime 3D Barrier Medium (CELLnTEC) for one day, before lifting the ASC-enriched patch to the air-liquid interface to induce cell differentiation for 10 days.

Chick chorioallantoic membrane model

Fertilized chicken eggs were incubated at 37 °C and placed with the smaller convexity pointing upward from ED1 (Embryo Development day) to ED4. At ED4, a hole was drilled through the smaller convexity pointing of the shell. At ED 7, the eggs were opened with scissors through the hole and the inner membrane to create a round window with approximate 1cm diameter. The developing chorioallantoic membrane was then irritated through creation of a micro-hemorrhage. With ASC in suspension, a silicon ring with 4 mm inner diameter was placed on the site of the generated hemorrhage. The ASC-enriched patched, fibroblast-enriched patches or control empty patches were deposited directly on the site of the generated hemorrhage. After implantation the window in the eggshell was covered with a paraffin film and placed in the incubator at 37°C. The number of vessels connexions to the patch were counted manually under a binocular loop.

Migration and tubulogenesis of HUVEC

HUVEC cells were purchased and cultured in complete endothelial cell medium 2 (both from Sigma). Migration of HUVEC was analyzed by using the endothelial cells migration assay (Sigma) according to the manufacturer's instructions. Briefly, HUVEC were starved for 15h in the endothelial cell medium 2 without serum and supplement and introduced in a Boyden chamber with at their bottom a hemi-permeable membrane coated with fibronectin or Bovine SerumAlbumin (BSA) used as a control. Migration through the fibronectin layer towards

supplement-free endothelial cell medium 2 conditioned 48 hours by ASC was measured by cell coloration (crystal violet) and extraction of the dye having migrated outside the Boyden chamber (via measurement of the absorbance of the extract at 540 nm). The migration was calculated as the difference between the absorbance with fibronectin and the absorbance with control BSA. For the tubulogenesis assay, serum/supplement-free endothelial cell medium 2 was conditioned for 48h with ASC. The analysis of tubular assembly of HUVEC was performed in the presence of each conditioned medium by using the angiogenesis assay kit (Abcam) according to the manufacturer's instructions. Briefly, HUVEC were plated in their conditioned medium on a fibronectin-containing gel for 24h, prior to cell coloration by a fluorescent dye and analysis of tubes via the Cytation 5 cell imaging reader (Agilent).

Statistical Analysis

Statistical analysis was performed using GraphPad Prism version 6.0 (Graphpad Software, La Jolla, CA). P-values less than 0.05 were considered statistically significant, and were indicated as follow: *: $p<0.05$; **: $p<0.01$; ***: $p<0.001$.

Supplementary Figure Legends

Figure S1. Generation of the ASC-patch

A) ASC derived from the abdominal fat and introduced within gelatin sponges and cultured during 7 days in air/liquid interface conditions to generate ASC-pacth. B) Hemalum/eosin coloration of an ASC-patch at day 0, immediately after ASC seeding. C) Hemalum/eosin coloration of an ASC/collagen gel composite 24h after ASC inclusion. B,C) images representative of 3 experiments.

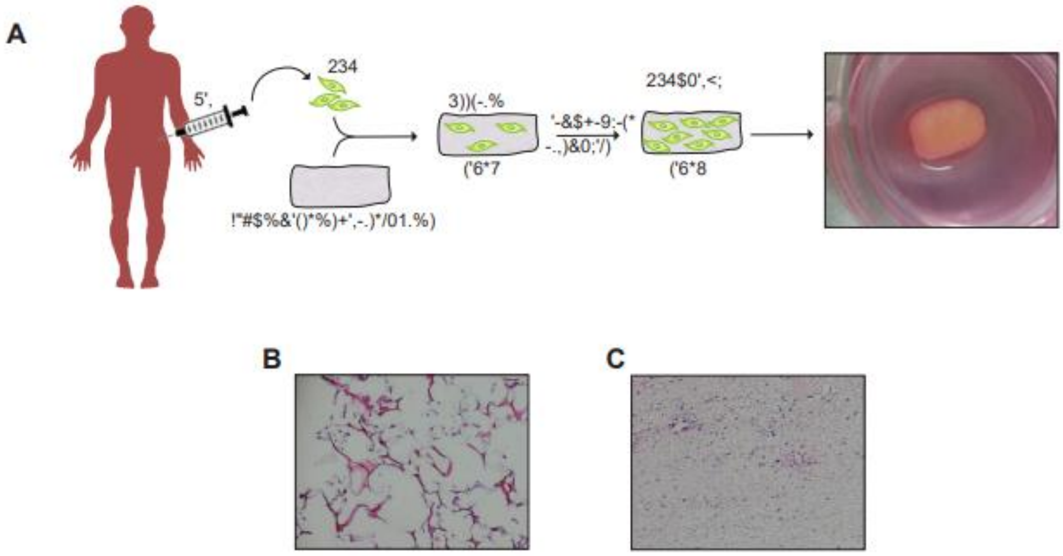

Figure S1

Figure S2. ASC in gelatin sponge regulate their regenerative transcriptome

The transcriptome of ASC-patches generated from 3 ischemic patients were assessed by microarray and compared to ASC from the same donors grown in monolayer. A) Hierarchical clustering of the two different conditions. B) Principal Component Analysis (PCA) of the microarray data of ASC-enriched patch (at day 7), ASC grown in monolayer (at day 7), freshly

isolated ASC, embryonic bodies (EB) and human fibroblasts (HF). C) Isolated and less represented clusters from analysis shown in Figure 2.

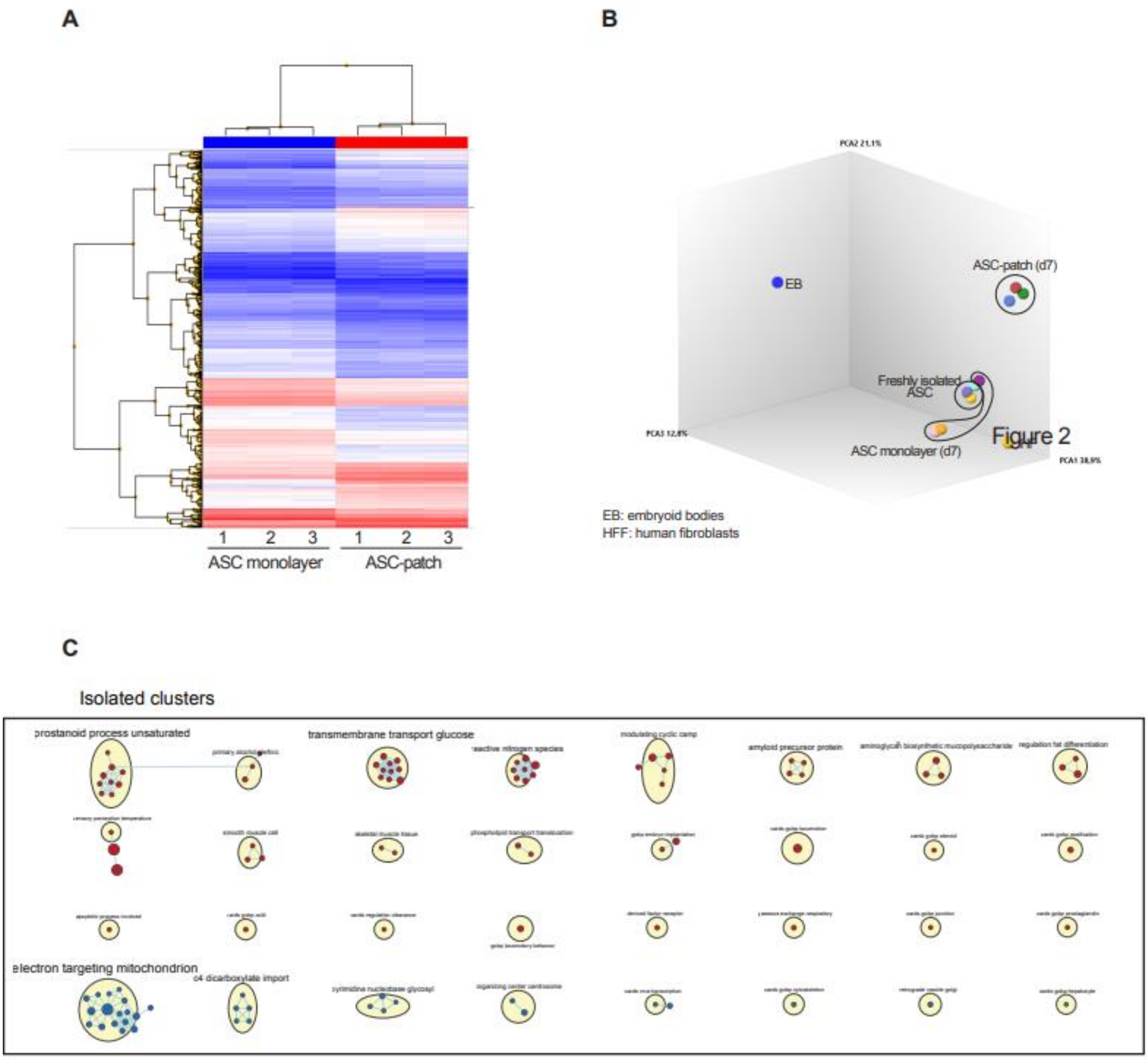

Figure S2

Figure S3. Healing factors concentrated in the ASC-patch supernatants

Quantification of the protein level of the indicated healing factors by bio-array in the supernatants of ASC-patches and ASC grown in monolayer.

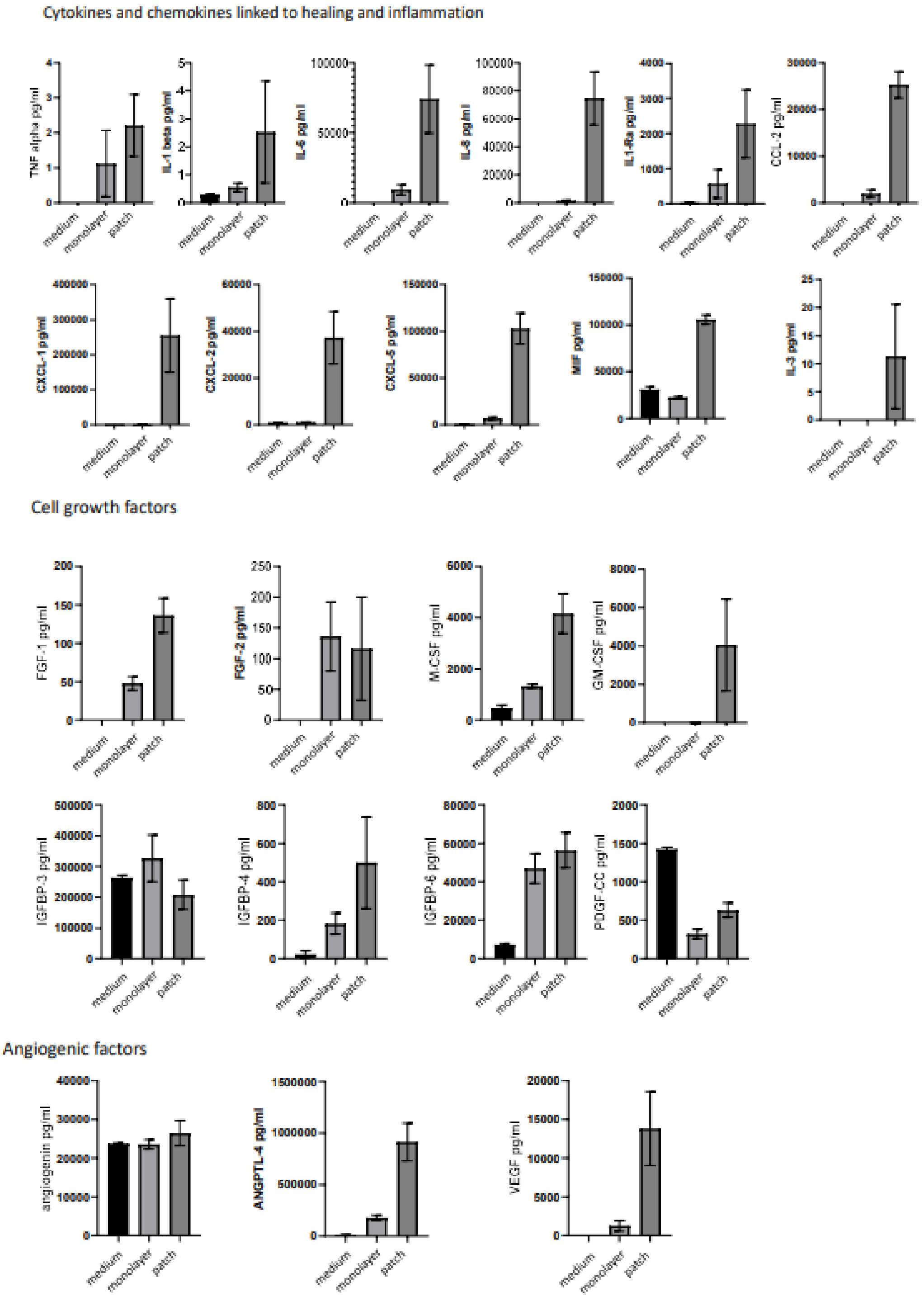

Figure S3

Figure S4. The acellular gelatin sponge by itself releases collagens and keratins.

Empty gelatin sponges were incubated for 24h in a serum-free medium, prior to a mass spectrometry analysis of the released proteome. Clustered protein according stringDB are shown. The experiment was performed in triplicates.

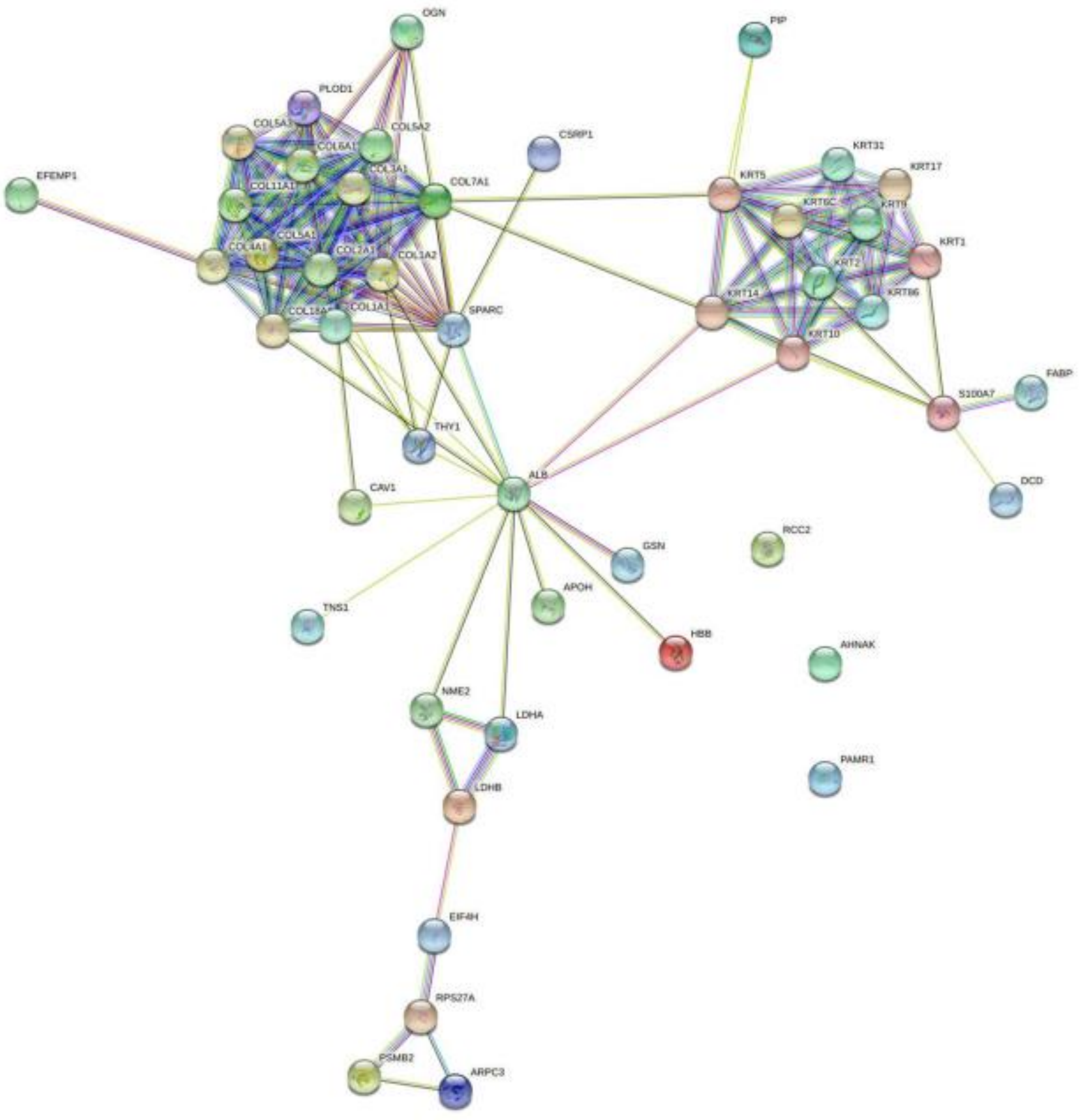

Figure S4

Figure S5. The ASC-patch promotes the growth on healthy fibroblasts and keratinocytes.

A) Autologous primary keratinocytes were seeded on the ASC-patch for 15 days in air-liquid interphase. Shown is a representative hemalun/eosin coloration (left), pan-keratin immunohistochemistry (middle) and BP-180 immunofluorescence (right). BP-180 refers to the alpha chain of type XVII collagen, indicating basal epidermal layer. B) Serum-free medium was incubated or not with ASC-patches generated from 3 different ischemic patient prior to its addition to human foreskin fibroblasts in monolayer. Cell viability was measured by trypan blue exclusion.

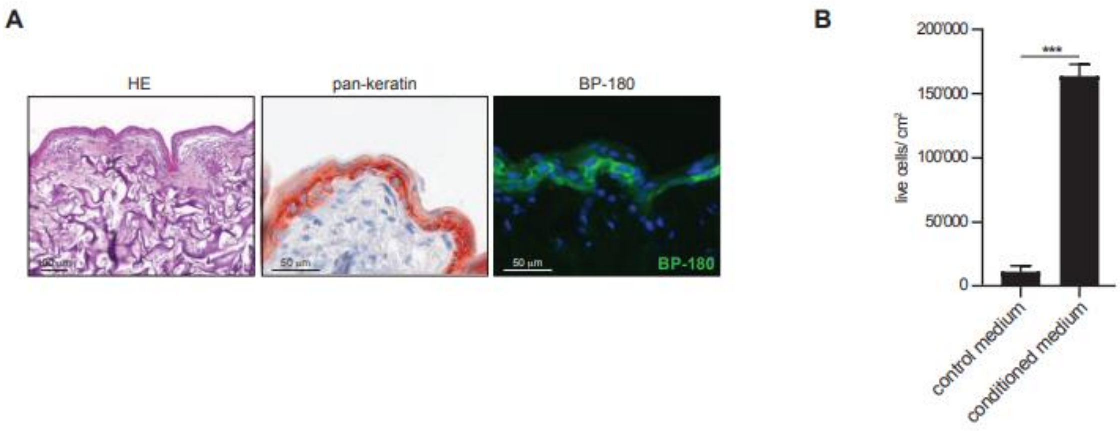

Supplementary Figure 5

Figure S6. The ASC-patch is characterized by a tissue-like structure and displays an enhanced regenerative transcriptome.

A) Macroscopic image of an ASC-patch prepared from rat ASC as colored by Hemalum/eosin. B-C) The transcriptome of 3 rat ASC-patches were assessed by microarray and compared to the same rat ASC grown in monolayer. B) Heatmap representation of the whole trascriptome of the two conditions. C) Metascape-based enrichment analysis using Gene Ontology

Biological Process gene set showing the most enriched pathways and processes identified within the significantly up-regulated (left) and down-regulated (right) genes (fold change>2, FDR<0.05) in the rat ASC-patch as compared to the rat ASC monolayer condition.

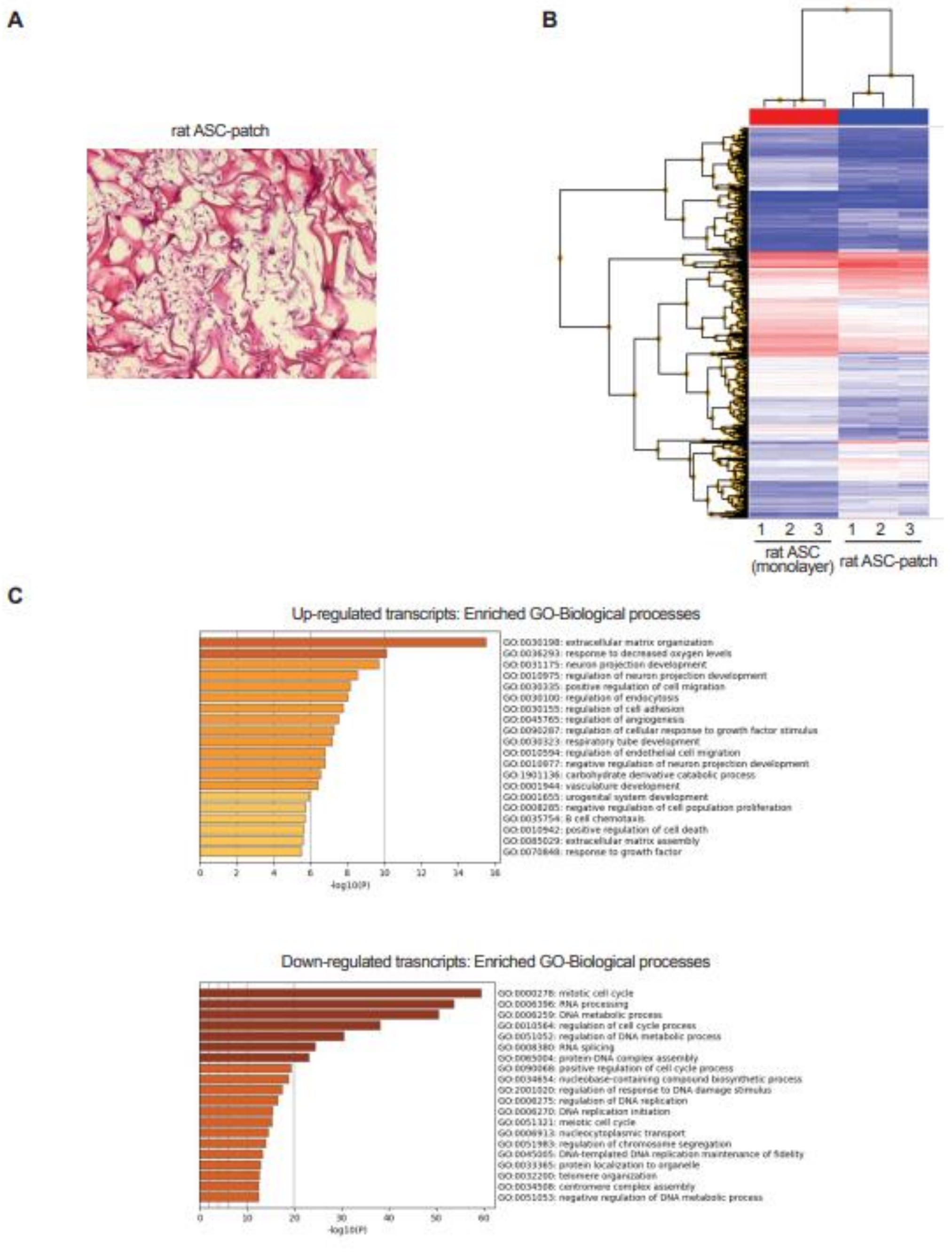

Figure S6

Figure S7. The ASC-patch did not stimulate ectopic tissue formation.

Rat ASCs stably transduced with firefly luciferase (FLuc) under the control of the ubiquitous promoter EFS (rASC EFS FLuc) were used to generate rar ASC-patches (FLuc-rASC-Patch). Intraperitoneal injection of D-luciferin allowed the monitoring of ASC in vivo by using the live imaging system IVIS Spectrum (Perkin Elmer). Representative image of a rat at day 8 (left) and 17 (right) post-treatment showing no ectopic tissue formation, as revealed by an absence of the luminescent signal outside the wound area.

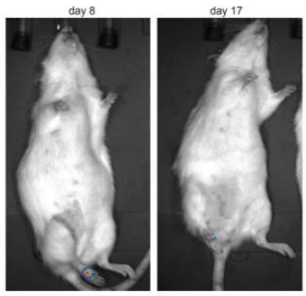

Figure S7
